# Notes and News

**Published:** 1905-10-07

**Authors:** 


### Notes and News.

The Gateshead Dispensary will receive £10,000 through the will of the late Councillor G. Gilhespy.

Five of the members of the Commission on the Feeble-minded have gone to America to study the arrangements for the care and control of the feeble-minded in that country.

The sum of £500 has been allocated to the Newsvendors' Benevolent and Provident Institution by Messrs. Gilbert W. Thomas and H. C. Scott, in the exercise of their discretion as executors of the will of the late Horace Harral, Esq.

The Odontological Society of Great Britain is prepared to receive applications for grants in aid of the furtherance of scientific research in connection with dentistry. Particulars will be supplied by the Scientific Research Committee, 20 Hanover Square.

A new hospital for Infectious Diseases was opened recently by the Duke of Northumberland at Newburn. The buildings consist of administration block of two stories, a typhoid pavilion, scarlet fever, observation wards, laundry, etc.—the whole accommodation being for 28 beds. The cost was £7,964, or just over £245 per bed.

Bile Beans for Biliousness were the subject of some strong remarks in the Law Courts of Edinburgh, when Lord Ardwall explained that these pills did not contain any special drug, as claimed by the vendors, but were an ordinary compound made by contracting chemists for them. The vendors are an unqualified chemist's assistant and a young stationer and printer.

If medical men in England would unite to obtain reasonable fees a satisfactory result would probably follow. An instance has occurred at Cholet, in France, where united action has been taken by the medical men resident there, as a benefit society was formed which secured 4½d. per consultation and 7d. per visit to the medical man. This scale was to apply to night visits and operations also.

A grand American bazaar is to be held in the Town Hall, Stratford, on October 26, 27, and 28, in aid of the West Ham and East London Hospital. The American Ambassador will perform the opening ceremony. The Duchess of Marlborough is President of the hospital, and is taking a very active part in furthering the efficiency of the hospital, and she will hold a stall at the bazaar and also write the "Book of the Bazaar."

As sanitary knowledge advances so does our choice of food become more limited. "Revelations" of objectionable methods of preparation are constantly causing us to reject some one or other item of diet, which probably has been regarded with much favour in the past. The latest warning is in relation to Smyrna figs. A correspondent in a contemporary writes from Smyrna pointing out that the sea water used by some of the packers in the packing of the figs comes from the bay which is polluted by the drainage of the city. As there is no means of selecting figs not so treated, Smyrna figs will be placed on the list of suspected foods.

The hospitals of Hull are very fortunate in having so active an executive committee of their Hospital Sunday Fund; owing to their activity there is no sign of hopeless apathy in the town, which is so conspicuous in the majority of urban communities. No effort is spared to excite interest in the cause for which the Fund works. The committee have issued 30,000 copies of a pamphlet entitled "A Guide to Sanitation." It contains an illustrated history of the various institutions existing at Hull for the benefit of the health of the population, interspersed with a useful exposition on health subjects. The cost of the pamphlet was defrayed by the advertisements is contains.

A strong appeal is made in the current number of the *Hospital Saturday Fund Journal* to trade unions and friendly societies to co-operate in an attempt to check the ravages of consumption by providing sanatoria for their members and by other means. A forcible argument in favour of such co-operation is the present heavy cost which tuberculosis entails upon friendly societies; for consumption being a chronic illness causes chronic expenditure. Thus in one court of the Ancient Order of Foresters, according to its secretary, eighteen members of the court had died in the past ten years. Of these eighteen deaths five were from consumption and thirteen were from other causes. But the amount of sick pay disbursed to these patients was £105 to the five consumptives, and £91 to the thirteen who died from other illnesses. In other words, the cost of the consumptive cases was three times that of the other cases. To this friendly society, which has a membership of over half a million, the deaths of members from phthisis entail a cost of more than £20,000 a year. There is every reason to suppose, on this account, that, apart from humanitarian motives, a rational expenditure on preventive measures would be a very sound financial undertaking.

